# A Temperature-Stable Cryo-System for High-Temperature Superconducting MR *In-Vivo* Imaging

**DOI:** 10.1371/journal.pone.0061958

**Published:** 2013-04-18

**Authors:** In-Tsang Lin, Hong-Chang Yang, Jyh-Horng Chen

**Affiliations:** 1 Interdisciplinary MRI/MRS Lab, Department of Electrical Engineering, National Taiwan University, Taipei, Taiwan; 2 Department of Electro-Optical Engineering, Kun Shan University, Tainan, Taiwan; 3 Department of Physics, National Taiwan University, Taipei, Taiwan; Texas A&M University, United States of America

## Abstract

To perform a rat experiment using a high-temperature superconducting (HTS) surface resonator, a cryostat is essential to maintain the rat's temperature. In this work, a compact temperature-stable HTS cryo-system, keeping animal rectal temperature at 37.4°C for more than 3 hours, was successfully developed. With this HTS cryo-system, a 40-mm-diameter Bi_2_Sr_2_Ca_2_Cu_3_O_x_ (Bi-2223) surface resonator at 77 K was demonstrated in a 3-Tesla MRI system. The proton resonant frequency (PRF) method was employed to monitor the rat's temperature. Moreover, the capacity of MR thermometry in the HTS experiments was evaluated by correlating with data from independent fiber-optic sensor temperature measurements. The PRF thermal coefficient was derived as 0.03 rad/°C and the temperature-monitoring architecture can be implemented to upgrade the quality and safety in HTS experiments. The signal-to-noise ratio (SNR) of the HTS surface resonator at 77 K was higher than that of a professionally made copper surface resonator at 300 K, which has the same geometry, by a 3.79-fold SNR gain. Furthermore, the temperature-stable HTS cryo-system we developed can obtain stable SNR gain in every scan. A temperature-stable HTS cryo-system with an external air-blowing circulation system is demonstrated.

## Introduction

The use of high-temperature superconducting (HTS) radio-frequency (RF) surface resonators has been proposed as a promising tool for magnetic resonance (MR) imaging due to its low impedance, which is below the critical temperature (Tc). Improvement of signal-to-noise ratio (SNR) using HTS surface resonators has been reported in various studies performed either on humans [1], [2], [3], small animals [2], [4], [5], or *in-vitro* samples [6], [7]. HTS thin films and HTS tapes are usually used as receiving coils. The present HTS thin films involve a high cost and complicated fabrication process that decrease their attraction and limit their large-scale application. Instead, commercialized bismuth-based HTS tapes, such as Bi_2_Sr_2_Ca_2_Cu_2_O_x_ (Bi-2223) and Bi-2212 tapes, have become potential choices for RF surface resonators in MRI. Grasso et al. [8] measured the quality factors (Qs) of surface resonators made with Bi-2223 tapes and observed only slightly fewer Qs than that of YBa_2_Cu_3_O_y_ (YBCO) films. Yuan et al. [9] investigated the Qs theoretically and verified them with experiments. These two investigations reported that HTS surface resonators have the potential to obtain a higher SNR for imaging than copper surface resonators. Cheng et al. [10] built a 5-inch HTS receiving surface resonator for a 0.21-Tesla (T) MRI system, and demonstrated a three-fold SNR improvement over an equivalent room-temperature copper surface resonator, which illustrates the feasibility of HTS surface resonators for MRI.

MR imaging using Bi-2223 surface resonators of 7 and 4 cm in diameter were studied [11]. A 1.5-fold signal-to-noise ratio (SNR) gain for kiwi fruit imaging and a 2-fold for a rat brain, both at 77 K, were obtained, compared with images obtained using a conventional copper surface resonator at 77 K. However, most cooling systems dedicated to MRI detection involved direct immersion in a bath of liquid nitrogen (LN_2_). LN_2_ bathes were contained in styrofoam vessels [12], [13], or in non-conductive Dewar vessels made of various non-conducting materials, such as PVC [14] and fiberglass composites (G10) [15], [16], [17]. The duration of such thermal insulation is approximately 1 hour for imaging samples. This is insufficient for a functional MRI experiment which lasts longer than 2 hours[18]. To improve the insulation duration, a temperature-stable cryo-system was proposed and designed in this study.

When developing the HTS cryo-system, this study employed for the first time glass to fabricate the cryostat. Glass was used because it can be a unibody that provides sufficient vacuum space for preventing thermal convection, and it is cheaper and easier to use compared with G10. The limited autonomy can be improved by supplying a LN_2_ stream through flexible pipes connected to a standard remote cryostat [19]. Another feature of the HTS surface resonator with a diameter of 4 cm is that it can obtain a larger field-of-view (FOV) and deeper image than Bruker cryoprobe with its resonator's diameter of 1 cm for small-animal MRI [20]. Finally, the MR proton resonant frequency (PRF) method [21] was employed to provide temperature information on the HTS cryo-system and to correlate the measured temperatures. The capability of a temperature-stable HTS cryo-system employed for an *in-vivo* rat brain experiment of two hours was demonstrated. This is also our first report on *in-vivo* rat brain images.

## Results

### MR thermometry with an imaging experiment

**Figure 1:**
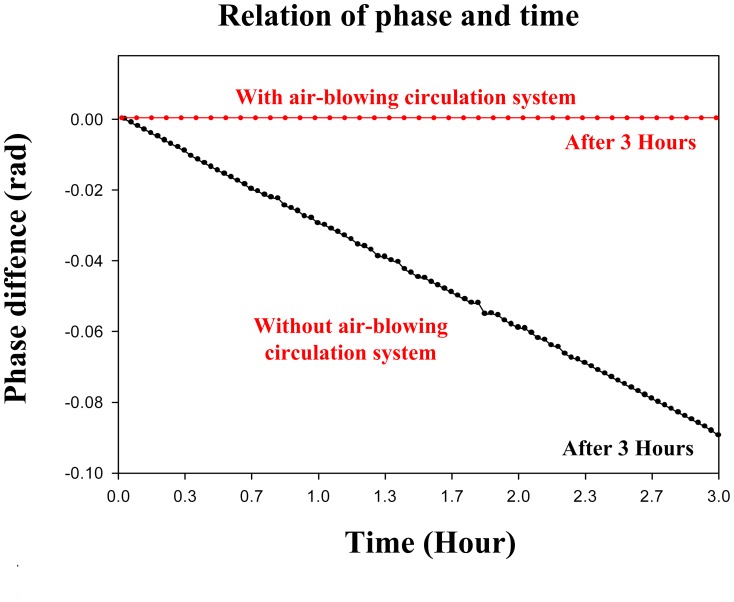
Temperature-to-phase curves with and without external air-blowing system.

First, the experiment was performed without an external air-blowing circulation system. After the localization and acquisition of conventional T1 and T2 images, the gradient echo sequence was repeated to acquire the phase data for temperature mapping. The temperature of the sample decreased from 22°C to 19°C after 3 hours of measurement. Figure 1 shows the time-to-temperature curve with the measured data of ΔP and ΔT, where ΔP is the change of phase difference in MR signal and ΔT is the change of temperature. The linear fitting curve was ΔP = γΔT−0.0013, and the PRF thermal coefficient γ was then determined as γ = 0.03 rad/°C, as shown in Figure 1 (Black line). These results were derived from the measured data of ΔP and ΔT. With this relationship, temperature variation can be derived by measuring the phase difference [21]. Next, the experiment was performed with an external air-blowing circulation system. It was found that the temperature of the sample was kept at 22°C during the 3-hour measurement, as shown in Figure 1 (Red line). A temperature-stable HTS cryostat was demonstrated.

### *In-vivo* rat brain imaging and experiments

**Figure 2:**
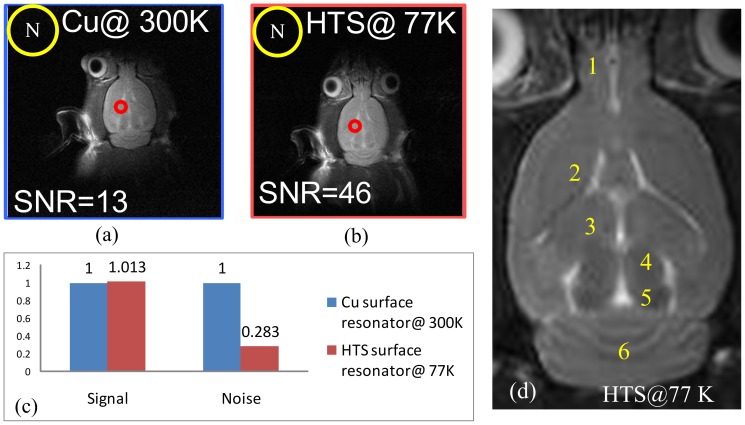
Rat brain images from HTS and professionally made copper surface resonators. (a) Image acquired from HTS tape surface resonator at 77 K. (b) Image acquired from copper surface coil at 300 K. (c) The SNR obtained using the HTS tape surface resonator was 46, 3.79 folds higher than that obtained using the copper surface resonator, which is 13 at 300 K. (d) Detailed *in-vivo* rat brain image acquired from HTS surface resonator at 77 K for 131 minutes from rat axial view. The structures are: 1) Olfactory bulb, 2) Caudate putamen, 3) Lateral ventricle, 4) Corpus callosum, 5) Dentate gyrus and 6) Superior colliculus.

The cryostat with an external air-blowing circulation system could maintain stable temperature for 3 hours. Then *in-vivo* rat brain images were examined. The unloaded quality factor *Q*_UL_(HTS) is 1330 while the loaded quality *Q*_L_(HTS) is 985 for a HTS surface resonator at 77 K. The value of *Q*_UL_ (Cu) and *Q*_L_ (Cu) are 300 and 155, respectively for a professionally made copper surface resonator at 300 K. The imaging time is 1 minute and 36 seconds. SNR between the cortical regions and background noise of both images were calculated to compare the performance of HTS and copper resonators. Rat brain images from HTS and professionally made copper surface resonators are compared in Figure 2. Figure 2(a) shows the image acquired from the HTS tape surface resonator at 77 K and Figure 2(b) shows the image acquired from the professionally made copper surface resonator at 300 K. As can be seen, the SNR of the HTS surface resonator at 77 K is 46. This is 3.79-fold higher than that of the professionally-made copper surface resonator, which is 12 at 300 K. However, the predicted SNR gain calculated using equation (1) was 3.76, which is slightly lower than the SNR gain of 3.79 obtained in the *in-vivo* rat experiment. The difference in SNR, as shown in Figure 2(c), is generally attributed to noises.

A further experiment for testing the duration capacity of the HTS cryo-system and a longitudinal *in-vivo* rat brain experiment were performed. The results are presented in Figure 2(d). As can be seen, the *in-vivo* rat brain structures from an axial view image acquired from the HTS surface resonator at 77 K in 130 minutes include the 1) olfactory bulb, 2) caudate putamen, 3) lateral ventricle, 4) corpus callosum, 5) dentate gyrus and 6) superior colliculus. The diameter of the HTS surface resonator is 4 cm so that the covered field could display an image from the olfactory bulb to the cerebella.

## Discussion

This study developed a temperature-stable HTS cryo-system, which could obtain a stable SNR gain in every scan. A HTS surface resonator with a diameter of 4 cm could obtain a larger FOV and deeper image than a Bruker cryoprobe with a resonator's diameter of 1 cm for small-animal MRI [20]. The advantages of a larger HTS surface resonator include better FOV and penetration depth. However, the smaller surface resonator of a Bruker cryoprobe has a higher SNR than a HTS surface resonator because of the resonator's diameter size. It should be noted that we do not need to compare it with a commercial system, because we cannot use the commercial system to image rat brain images.

### MR thermometry in MRI

Thermal insulation of the cryo-system is a crucial issue in HTS cryo-systems for *in-vivo* studies. The PRF method was employed to determine phase changes from the temperature-dependent change at resonant frequency [21]. The MR thermometry using the PRF method was integrated into the HTS experiment to monitor temperature changes of rat samples. The MR thermometry was evaluated by correlation with independent fiber-optic sensor temperature measurements [21]. The fiber-optic sensor temperature was acquired from the surface of the cylindrical phantom and close to the PRF acquisition slice. A previous study used fiberglass material to fabricate the LN_2_ container [20]. The PRF thermal coefficient γ was 0.1181 rad/°C and the temperature could be decreased from 11.5°C to 6°C in 20 minutes.

Here we presented a temperature-stable cryo-system and our PRF thermal coefficient γ was 0.03 rad/°C. The body temperature of the rat subject and its breathing condition were monitored during the HTS experiment. The results obtained reveal the stability of the cryo-system with an external air-blowing circulation system. It should be noted that the cryo-system remained temperature-stable until the LN_2_ supply is exhausted. In our study, the temperature could be maintained for 5 hours. In further experimentation, the PRF method may be combined with segmented echo planar imaging (EPI) principles and echo-shifting principles to improve scanning speed. For the above method, a standard deviation of less than 1°C for a temporal resolution below 1 second and a spatial resolution of about 2 mm are feasible for a single-slice immobile tissue [21].

### Rat brain imaging experiment

*In-vivo* rat brain images for the imaging time of about 2 hours, which were obtained by the HTS surface resonator, are presented. Owing to the high performance of the cryostat, the temperature can be kept stable. The results show that for anatomical images, a HTS surface resonator at 77 K provides a 3.79-fold SNR gain over a professionally made copper surface resonator at 300 K. Experimental results are in agreement with the predicted ones, and the difference between the predicted SNR gains and measured SNR gains is 0.8%. The results also demonstrate higher *in-vivo* image quality obtained using the HTS surface resonator. With the improved SNR of the HTS surface resonator at 77 K, the imaging time can be shortened to 1/(3.79)^2^ while mataining the same SNR of the copper surface resonator at 300 K. With this stable HTS cryo-system, the HTS surface resonator may be further used in a functional MRI.

In addition, the imaging time can also be significantly reduced by parallel processing with phased array resonators [22]. The potential benefits justify the development of a practical HTS surface resonator for imaging systems despite considerable technical difficulties and challenges involved in the usage of a cryo-system and volume resonators with different shapes (e.g., solenoiid, saddle or birdcage [23]).

### Discussion on longitudinal experiment

The capability of this temperature-stable HTS cryo-system for longitudinal *in-vivo* rat brain applications is demonstrated. Regarding the thermal insulation issue, the free space for thermal insulation on an animal HTS cryostat is typically limited due to the gradient with an inner diameter of 12 cm and the size of target sample.

It is important to choose adequate material for cryo-system design to improve thermal insulation and prevent samples from freezing. However, it is impossible to achieve complete thermal insulation. This is usually due to incomplete vacuum insulation. In previous studies, styrofoam and G10, which are popular materials for fabricating a cryostat, were used [24]. The G10 cryostat was insulated with vacuum layers and the styrofoam cryostat was only insulated by styrofoam alone. However, there are still some problems in clinical practices due to the complexity of manufacturing a G10 cryostat, while the thermal insulation of styrofoam is bad. With these two materials, a HTS surface resonator can only be applied in in-vitro studies and the applications are limited. A comparison of cryostats made from styrofoam, G10 and glass are listed in Table 1.

**Table 1:** Comparisons of styrofoam, G10 and glass.

	Insulation	Vacuum layer(torr)	Minimum thickness(mm)
Styrofoam	Low	No	5
G10	high	10^−3^−10^−5^	5
Dewar made of glass	High	10^−3^−10^−5^	2

The stability of a HTS cryostat has also been considered as another critical issue in design. Typically, the resonance frequency of a cooled HTS surface resonator shifts slightly as LN_2_ is fed into the cryostat. Therefore, it needs a period of time to stabilize the resonance frequency under the critical temperature when cooling a HTS surface resonator. The SNR can be improved with a high-quality HTS surface resonator, suggesting that a HTS surface resonator is a potentially helpful diagnostic tool for MRI imaging in various applications. Further applications of a functional MRI and dynamic contrast-enhanced (DCE) MRI are under investigation to test the applicability of this HTS cryo-system in a 3T system [25].

## Materials and Methods

### Estimation of SNR Gain Using Quality Factor

The Qs is a highly crucial parameter when estimating the resistive loss of RF resonator. The frequency responses of surface resonators and the values of Qs were measured using a Hewlett Packard-8751A Network Analyzer set in the S_11_ (the reflection coefficient) mode. The S_11_ response is basically the ratio of the reflected power to the total transmitted power given by the network analyzer via the signal pick-up resonator [11]. A study on the values of Qs for a Bi-2223-based LC resonator was reported in [8]. The Qs of the resonant circuit are then defined as 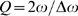, where 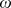 is the resonant frequency and Δω is the bandwidth of the resonant frequency at -3dB [26]. Assuming that the preamplifier noise voltage is negligible as compared with that of the resonator and the sample, the theoretical SNR gain of the HTS surface resonator at 77K over the professionally made copper surface resonator at 300 K can be estimated using equation (1) [2], [27].
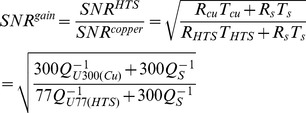
(1)where SNR^HTS,77K^ and SNR^copper,300K^ are respectively the SNRs obtained from the HTS surface resonator at 77 K and the copper resonator at 300 K; T_cu,300K_ and T_HTS,77K_ are the temperature of the copper resonator and HTS surface resonator, which are 300 K and 77 K, respectively; R_cu,300K_ and R_HTS,77K_ are the resistance of the copper resonator at 300 K and the resistance of the HTS resonator at 77 K, respectively; Q_U300_(Cu) and Q_U77_(HTS) are the unloaded Qs at 300 K and 77 K, respectively; and 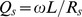 is the sample's Qs.

### Hardware

MR experiments were performed on a Bruker Biospec 3T MRI system (Bruker Biospin, Ettlingen, Germany) with an inserted gradient, for which the maximum gradient strength was 200 mT/m and the inner diameter was 12 cm.

**Figure 3:**
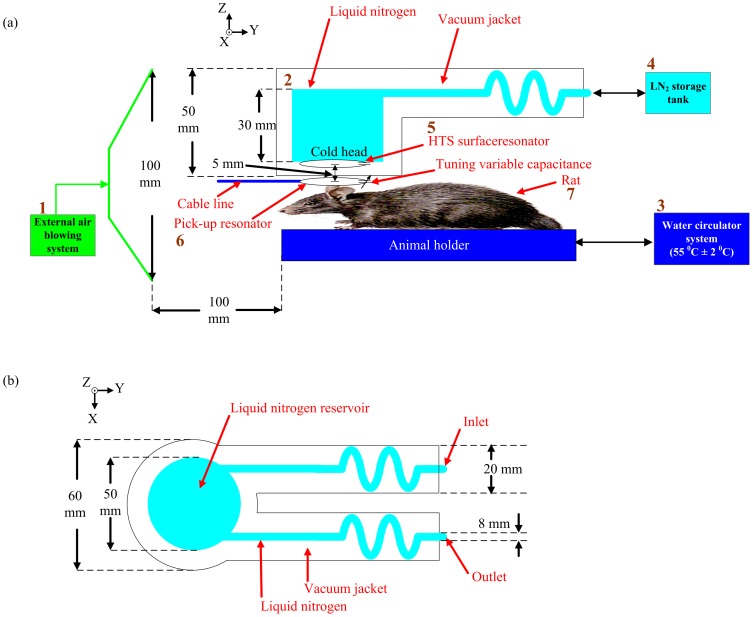
The whole view of the HTS cryo-system. (a) Lateral view of HTS cryo-system, including external air blowing system, cryostat, water circulation system, HTS surface resonator, pick-up resonator, sample (rat) and LN_2_ storage tank. (b) Schematic drawing of the cryostat.

Figure 3(a) shows the lateral view of the HTS cryo-system, including the external air-blowing system, cryostat, water circulation system, HTS surface resonator, pick-up resonator, sample (rat) and LN_2_ storage tank. The external air-blowing circulation system is placed in the MRI scan room, as shown in Figure 3(a). The external air-blowing circulation system blew fresh air through the gradient and the rate of air flow was 0.16 m^3^/s. The temperature of the fresh air from the front of the gradient was measured to be 22°C. For cooling HTS surface resonators, an entirely non-magnetic and non-metallic cryostat was designed for MR experiments. The cryostat, including the circulating cold head, was manufactured with borosilicate glass (Pyrex glass). The glassed cryostat used for MRI experiments was placed inside the gradient bore with the diameter of 12 cm. The cryostat was placed on the acrylic support and the rat was placed under the cryostat. The schematic drawing of the cryostat is shown in Figure 3(b). The cryostat has a cylindrical LN_2_ cold head connected to a U-shaped pipeline inside the vacuum jacket. The dimensions of the cylindrical LN_2_ cold head in the cryostat were about 50 mm in diameter and 30 mm in height. The cryostat with a vacuum space was designed to provide thermal insulation for the longitudinal experiment. The vacuum pressure was kept below 10^−7^ torr to reduce thermal convection. A LN_2_ cold head located inside the vacuum jacket was in contact with the HTS surface resonators, as shown in Figure 3(a). The LN_2_ cold head provides cooling to the HTS surface resonator when the LN_2_ circulates around the LN_2_ cold head. An adhesive Eccobond 286 A/B (Krayden, inc.) was employed to connect the LN_2_ cold head with the HTS surface resonator. Eccobond 286 A/B is a two-component, thermally conductive, room-temperature-cured epoxy adhesive and its average shear strength of thermal stress tests are 38.9 MPa at 293 K and 95.4 MPa at 77 K. Eccoband 286 A/B is non-magnetic; hence, the adhesive has no effects on MRI images. The imaging sample (rat) was placed in the animal holder, which is warmed by a water circulation system to keep the temperature of the animal at 37.4°C (rat). During the rat experiment, the water circulation system can be adjusted by changing the water temperature. LN_2_ was fed from the LN_2_ storage tank through a rubber tube. A LN_2_ tank with a self-pressurized transferring system (TP60, Air Liquide, France) was connected to the cryostat for pumping the LN_2_, which can be transferred out of the tank by the high pressure inside the tank, and the LN_2_ transferring rate was controlled by a pressure-controlled valve. The LN_2_ pathway inside the cryostat forms a U-turn, one side of the U-shaped pathway is the inlet while the other side is the outlet.

The separation between the HTS/copper surface resonator and sample is about 5 mm. The surface transceiver resonators were fabricated using the HTS tape material, Bi-2223 tape (Innova Superconductor Technology Co., Ltd., Beijing, China). The wires, with a multi-filamentary structure, show a critical temperature of 110 K and an engineering critical current density greater than 9000 A/cm^2^ at 77 K. The thickness and width of the raw HTS tape are 0.23 mm and 4.1 mm, respectively, with a 10 µm-thick tin alloy sheath to provide mechanical support for the HTS composition. The HTS surface resonator was wound into a circular shape and connected in series with a high-Qs capacitor (American Technical Ceramics, Hungington Station, NY, USA) to form an LC resonating circuit.

The professionally made copper surface resonator and pick-up resonator were constructed using a copper cable (Hitachi, Japan), which was made of 99.9999% (6N) purity copper, and a high-Qs capacitor (22 pF and Qs≈1000 at 125 MHz, American Technical Ceramics, NY, USA). It was non-magnetic and directly soldered at both ends. The diameter of the copper cable was 1.5mm.

**Figure 4:**
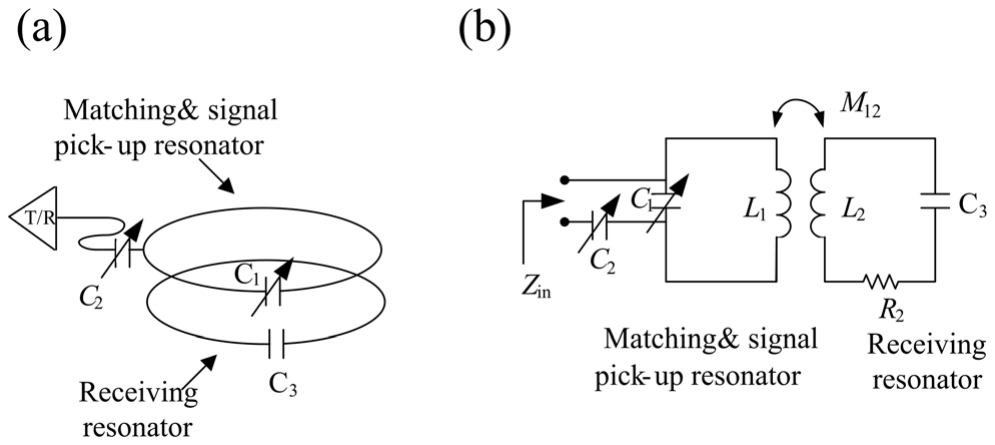
Concept of inductive coupling method. (a) Matching and signal pick-up coil and receiving coils. Two additional capacitors were soldered with the coupled coil to tune and match the center frequency to 125.3 MHz. (b) The equivalent circuit of the inductive coupled design shows that RF signal is picked up using mutual inductance coupling.

RF signal transmission and reception was accomplished using inductive coupling [11], as shown in Figure 4. Figure 4(a) shows the geometry of the receiving resonator and pick-up resonator, indicated respectively by the lower and upper circle. The diameter of both the receiving resonator and the pick-up resonator were 4 cm. The same pick-up resonator was used for both HTS and copper receiving resonators. Figure 4(b) shows the equivalent circuit of the inductive coupled design in which the RF signal is picked up by mutual inductance coupling. Furthermore, two trimmer capacitors C_1_ and C_2_ (Voltronics Corp., NJ, USA) were used in our study. C_1_ was soldered to the pick-up resonator for tuning the resonator system to the resonant frequency of 125.3 MHz, while C_2_ was soldered to the pick-up resonator for matching the resonator system to the standard preamplifier of 50 ohms. All the resonators were tuned to 125.3 MHz and the frequency response was measured on a vector network analyzer (Hewlett Packard-8751A, USA). It should be noted that a change on the matching the resonator system can be seen if the HTS/copper receiving resonator is removed. It should also be noted that the pick-up resonator was outside the cryostat and placed under the cryostat.

To compare between the image quality the copper surface resonator and that of the HTS surface resonator, the geometry of both resonators were set as a single-loop surface resonator (4 cm in diameter). The two resonators were both kept at the same distance from the samples and placed at the same position inside the cryostat. The power setting was determined for each individual surface resonator by stepping through a range of transmitting attenuation values in the pre-scans.

All the procedures of the animal experiments comply with the Guidelines for Care and Use of Experimental Animals established by the Institutional Animal Care and Use Committee (IACUC) at National Taiwan University College of Medicine and College of Public Health. All the experiments are performed with Sprague Dawley (SD) rats, kept in a 12-h dark/light cycle environment at a temperature of 25°C with food and water. Adult SD rats, weight 250–350 g, were used for this study. All animals were initially anesthetized with 3% isoflurane in a 1/1 Oxygen/Air mixture and injected with atropine (20 µg/kg) to avoid excessive amount of salivation [28]. The animals were secured on a custom-made holder. Respiration rate was monitored with an Invivo pulse oximeter 4500 MRI (Invivo Research, FL USA); rectal temperature was monitored with a Fiberoptic thermometer FL 2000 (Anritsu meter, Tokyo, Japan); heart rate was monitored with an Invivo pulse oximeter 4500 MRI (Invivo Research, FL USA). All the physiological data were stored in a computer.

### Imaging experiments

The first sample was a cylindrical phantom, which is 35 mm in diameter and 50 mm in height, filled with 2×10^−5^ mM Gadolinium (Gd) solution and placed under the cryostat. The total volume of the phantom is 61.25 ml and the phantom is entirely covered in the surface resonator area. To record the absolute temperature variation, an optic-fiber thermoprobe was fixed onto the surface of the phantom and the temperature was recorded every minute during the experiment. One horizontal slice at 1 cm below the sample surface was imaged to provide information on the internal temporal temperature variation. To reconstruct temperature mapping, the PRF method [21] was performed after the phantom was positioned in the center of the magnet for 180 minutes. The parameters of the PRF sequence are listed in Table 2. A total of 90 scans were acquired to reconstruct the temporal temperature change. Phase images were reconstructed to map the temperature change using the MR PRF method. The phase image acquired at the beginning serves as the reference phase and the phase difference (ΔP) at each interval can then be derived by subtracting the phase from the reference. Fitting the correlation between temperature difference (ΔT) and ΔP yields the PRF thermal coefficient.

**Table 2:** Parameters of sequences in MR PRF, *in-vivo* rat brain and longitudinal experiment.

Experiment	Sequence	TR (ms)	TE(ms)	Image matrix	FOV (cm^2^)	Total scanning time (min)	Flip angle(^0^)	Slice thickness (mm)
MR PRF	Conventional gradient echo sequence with spoiled gradient	21.7	7	128×128	10×10	180	15	5
Rat brain	Fast spin-echo sequence	3506	62	256×256	6×6	1.5	0	1.24
Longitudinal experiment.	Fast spin-echo sequence	3506	62	256×256	6×6	130	0	1.24

In the *in-vivo* animal study, rat brain imaging was carried out. The SNR gains of the copper resonator and the HTS resonator of the same geometry were compared. The total imaging time was 1 minute and 36 seconds. With the stable cryostat, the rat could be kept alive in a comfortable situation. Next, the longitudinal experiment was performed to acquire multiple rat brain images. The parameters of the sequence used in in-vivo rat brain and longitudinal experiments are listed in Table 2.

In a conventional image, the signal is measured by the mean value of pixels within the region of interest (ROI). The usual size of such an image is 0.5 cm by 0.5 cm. Standard deviation (STD) of background noise is measured using the largest possible ROI (avoid ghosting/aliasing or motion artifact regions). The SNR of an image is calculated as the ratio of the mean signal to the standard deviation of the background noise. SNRs were calculated to compare the performance of HTS surface resonator and professionally made copper surface resonator.
